# Supplementary material for: Ni-Catalyzed Enantioselective Intramolecular Mizoroki–Heck Reaction for the Synthesis of Phenanthridinone Derivatives
Source: J Org Chem. 2023 Jun 15;88(13):8203–26. doi: 10.1021/acs.joc.3c00202 (PMC10337041; doi:10.1021/acs.joc.3c00202)

DR-107-112 6-F iquinox 1H
PROTON CDCl3 {D:\nmrusers\malachowski} BM 5

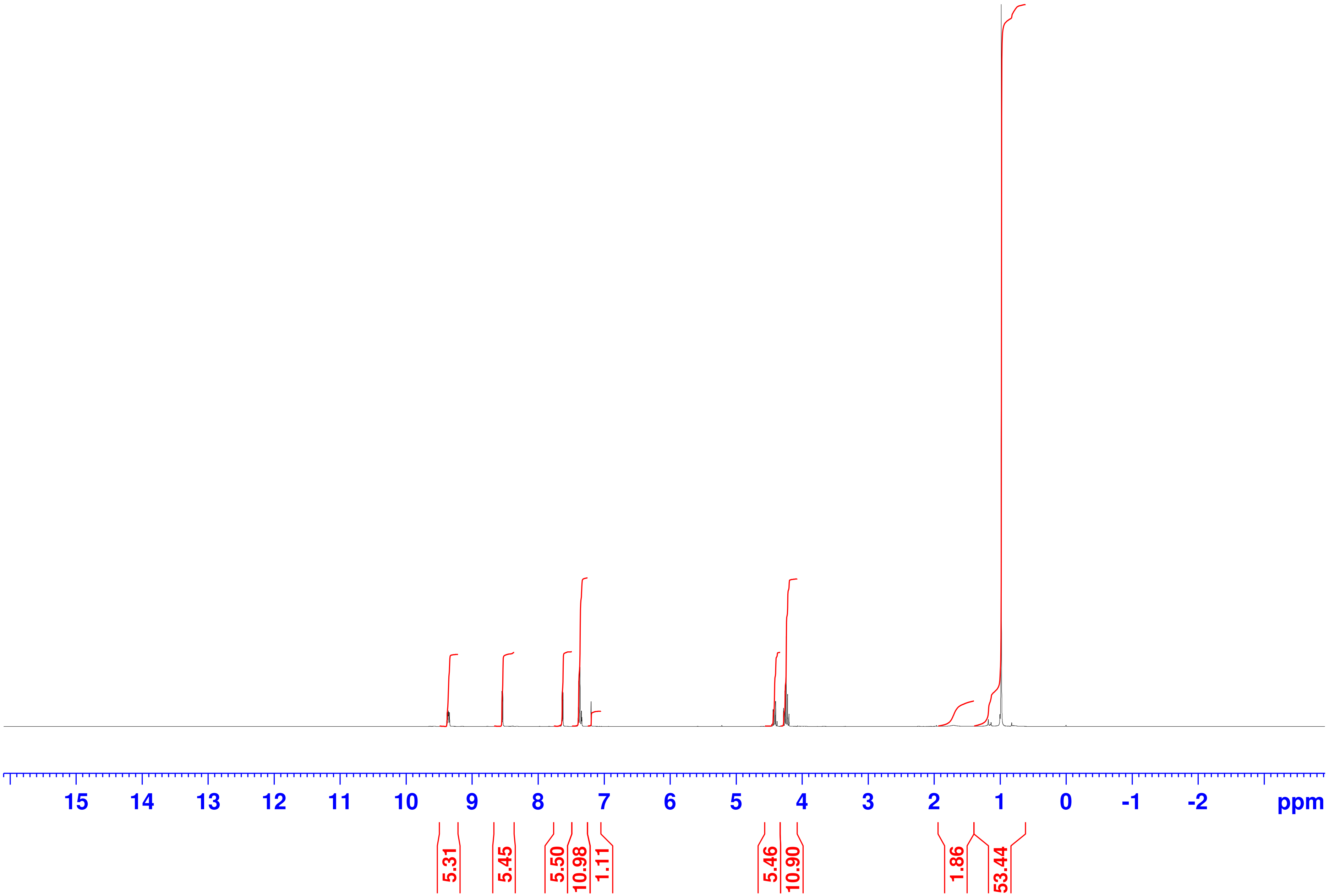

Supplement: Supplementary file 2 — jo3c00202_si_002.zip [file jo3c00202_si_002.zip › Chiral ligands/L26 6F-tBu-iQuinox/1H 6F-tBu-iQuinox L26/1/pdata/1/email_DR-107-112 6-F iquinox 1H_1_1.pdf]
